# Irisin at the crossroads of inter-organ communications: Challenge and implications

**DOI:** 10.3389/fendo.2022.989135

**Published:** 2022-10-04

**Authors:** Renqing Zhao

**Affiliations:** College of Physical Education, Yangzhou University, Yangzhou, China

**Keywords:** irisin, exercise, brain function, heart diseases, bone, liver

## Abstract

The physiological functions of organs are intercommunicated occurring through secreted molecules. That exercise can improve the physiological function of organs or tissues is believed by secreting myokines from muscle to target remote organs. However, the underlying mechanism how exercise regulates the inter-organ communications remains incompletely understood yet. A recently identified myokine–irisin, primarily found in muscle and adipose and subsequently extending to bone, heart, liver and brain, provides a new molecular evidence for the inter-organ communications. It is secreted under the regulation of exercise and mediates the intercommunications between exercise and organs. To best our understanding of the regulatory mechanism, this review discusses the recent evidence involving the potential molecular pathways of the inter-organ communications, and the interactions between signalings and irisin in regulating the impact of exercise on organ functions are also discussed.

## 1 Introduction

It has long been suspected that physiological functions are mediated through the inter-organ communications (1–3), and such communications mainly occur through secreted molecules. The muscle, for example, is known as a motor organ, but compelling evidence has implied that it is also an endocrine organ, secreting hundred proteins (known as myokines) during exercise that enter circulatory system and function as endocrine modulators mediating the adaption of organs and tissues to exercise (4). Obesity and insulin resistance are regarded as chronic inflammation diseases; endurance exercise is effective in improving obesity and insulin resistance by stimulating muscle to secret myokines promoting energy metabolism and alleviating inflammation status (2, 5, 6). Similarly, brain, heart, bone, and liver also are responded to exercise training *via* secreted myokines (7–12).

Since the identification of the novel myokine, fibronectin type III domain-containing protein 5 (FNDC5), this link between organs gains its new molecular evidence (5, 12–14). The expression of *Fndc5* is regulated by peroxisome proliferator-activated receptor γ coactivator 1α (PGC1α) that acts as a transcriptional co-regulator produced by exercising muscle (5). It has gained great attention in regulating the physiological and pathological processes of organs in the condition of health and diseases recently. It involves the process of inter-organ crosstalk between muscle, bone, liver, heart, and brain, and new evidence on the molecular signalings engaged in the communications has been proposed recently (1, 10, 12, 13, 15, 16). The primary goal of this review is to best our understanding of the signaling pathways for irisin regulating the inter-organ communications in health and diseases, and also to provide an approach to understanding the adaptations of organs to exercise

## 2 Physiological and biological aspects of irisin

### 2.1 General characteristics of irisin

**Figure 1:**
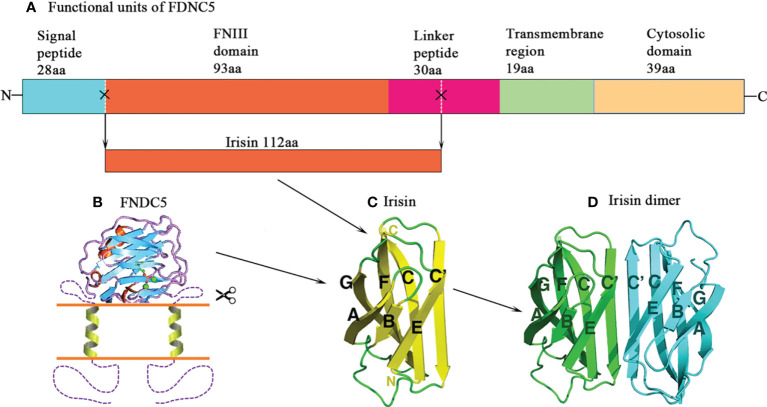
Structure of irisin. **(A)** Schematic structure of mouse fibronectin type III domain-containing protein 5 (FNDC5) protein. **(B)** A model of transmembrane FNDC5 and the linker that locates between the FNIII domain and the transmembrane helix. The N-terminal signal sequence is proposed to be cleaved. **(C)** Structure of irisin. It is a proteolytic product of FNDC5. The irisin domain is connected to a short transmembrane region, which is followed by the cytosolic region. **(D)** The diagram of the irisin dimer. The C’ strands pair to form an eight-strand beta sheet in the dimer.

About twenty years ago, two research groups independently reported a novel gene that was expressed in rodent skeletal muscle, heart, brain, and other tissues (17, 18). One decade later, this gene was named *Fndc5*, one of the target genes of PGC1α, a transcriptional co-regulator usually secreted by exercising muscles (5). FNDC5 is highly expressed in excising or genetically overexpressing PGC1α (*Ppargc1a*) mice, and the medium of cultured myocytes form mice overexpressing *Fndc5* induces the upregulation of uncoupling protein (*Ucp*) *1* mRNA in adipocytes, which is a pivotal gene promoting brown adipose tissues (BAT). Moreover, irisin also promotes mitochondrial biogenesis, another hallmark of fat browning, subsequently upregulating mitochondrial DNA contents (mtDNA) and mitochondrial biogenesis-associated genes, including mitochondrial transcription factor A (TFAM), nuclear respiratory factors 1 and 2 (NRF1 and NRF2) and UCP1 in human adipocytes (19, 20). So far, numerous studies have confirmed the role of FNDC5 in regulating energy metabolism (5, 12, 14). In the meantime, a novel myokine–irisin has been found; it comprises of 112 amino acid (aa) and is derived from FNDC5 by cleaving at the 30th and 142th aa sites by proteolytic enzymes (**Figure 1**) (5). It is proposed that the secreted irisin involves in the positive effect of exercise on metabolism through browning of adipose tissue. Thereafter, thousand papers have been published to explore the associations of FNDC5/irisin with a variety of exercise protocols and pathophysiological situations, as well as *ex vivo* and *in vivo* influence of recombinant irisin (r-irisin) treatment on various cells and different rodent models.

### 2.2 Detection of irisin

The construct of irisin contains the classical FNIII region and the linker (**Figure 1**) (21). Albrecht et al. (22) measured non-glycosylated r-irisin by performing a band with the molecular weight (MW) of 13 000, close to 12 300 for this molecule, and glycosylated irisin running a band with the MW of 20 000. There are mainly 3 methods to measure secreted irisin, *i.e.* Enzyme-Linked Immunosorbent Assays (ELISA), Western Blot, and Mass Spectrometry (MS). Among those approaches, MS is recognized as “gold standard” for measuring the protein levels (23). To date, the widely recognized reference levels for blood irisin were 3~4 ng/mL (24) and 0.3 ng/mL for humans and mice (14), respectively.

However, for measurement of irisin with Western blot methods, only a small number of studies reported the bands approximate to the predicated value for deglycosylated irisin (13 000) in rodent and human circulation (24–26). The most confusion concerning the detection of irisin with Western blot methods is that the reporting of bands of MW shows much divergent values, indicating that the anti-body used are different between the reports. Huh and colleagues (27) first determined circulating irisin using ELISA and reported a level of 113.1 ± 20.6 ng/mL among a population of common women. Afterward, De Meneck et al. (28) reported that children with overweight/obesity showed higher circulating irisin levels (143.1 ng/mL) than those with normal weight (75.2 ng/mL). This began a period of testing circulating irisin in various conditions (29–31). The most hallmark of those studies is the extreme values reported, varying from 40 pg/mL (30) to 14.78 µg/mL (32). No specific antibodies for irisin may account for the divergent reports.

Quantitative MS is conducted *via* a novel approach with the combination of detecting specific peptides for irisin and adding quantified dose of marked peptides to the sample which is applied to measure the concentrations of unmarked irisin within the sample by comparing the peak heights or areas. By labeling both FIQEVNTTTR and DSPSAPVNTVR (N-terminus), Jedrychowski et al. (24) reported the circulating irisin levels about 4 ng/mL in human samples. They also reported a circulating concentration of 4.3 ng/mL for irisin in the group with aerobic exercise training. In a murine serum sample, Albrecht et al. (33) measured irisin levels about 0.6 ~ 0.9 ng/mL. Before conducting MS measurement, proteins, such as albumins and immunoglobulins, in the blood samples must be removed that results in divergent amount of proteins left for measurement, which could lead to different irisin levels reported between studies.

### 2.3 Regulation of irisin secretion

Irisin is expressed almost in whole tissues and organs of rodents and humans; it presents in the liver, heart, kidney, brain, skin, muscle, retina, and pineal gland in rodents (17, 34–38), and also appears in human liver, pancreas, stomach, cerebrospinal fluid, serum, saliva and urine (39–42). Varied human tissues express diverse concentrations of irisin, with the highest amounts of irisin detected in skeletal muscle, proceeded behind by other muscle-contained tissues. Adipose tissue expressed a level of irisin almost hundred times lower than that of muscle (27, 43, 44). The secretion of irisin is affected by a number of factors, including exercise, cold, diet, drugs, and pathological situations (45), among which exercise is the strongest stimulator for promoting irisin expression. Exercise led to about threefold elevation of *Fndc5* mRNA levels in murine muscle compared with that of controls. Additionally, the expression of *FNDC5* mRNA in muscle was approximately twofold higher in adult receiving endurance training than that without exercising individuals (46). White adipose tissue (WAT) constitutes an alternative source of irisin contributing approximately one third total levels of circulating irisin, and the secretion of irisin in white adipose tissue significantly increases in response to exercise training (47).

## 3 Irisin-brain communication

### 3.1 Irisin and brain function

Exercise has many positive effects on brain health, such as reducing the risks of dementia, depression and stress, restoring or maintaining cognitive function and metabolic control (1, 48, 49). The facts that brain not only controls the action of muscle during physical exercise, but it senses and responds to proteins secreted from muscle during exercise, indicating a direct crosstalk between muscle and brain. Accumulating evidence has confirmed muscle as an endocrine organ, releasing myokines (such as cathepsin B and PGC1α) into blood to mediate the function of remote organs (4, 9). Since identified as a novel myokine, irisin has gained a lot of interests for its beneficial effects in regulating neuronal generation, synaptic plasticity, inflammation, as well as cognition. In this section, we will discuss the evidence on the mechanism that modulates the communication between irisin and brain function in physical and pathological conditions.

The expression of irisin in the cerebrospinal fluid (CSF) of humans has been detected by both Western blot and MS (17, 50). Moreover, many regions of brain express FNDC5, such as cerebellar Purkinje cells (5, 17, 18), the hypothalamus (51), and hippocampus (52). *FNDC5* mRNA is found to be up-regulated in differentiated human embryonic stem cells (hESCs) and reaches the highest expression level in neural cells (50, 52). Additionally, during the formation of neuronal precursors, *Fndc5* overexpression resulted in elevated glial fibrillary acidic protein (GFAP), brain-derived neurotrophic factor (BDNF), Neurocan, and Microtubule-associated protein 2 (Map2). Contrarily, *Fndc5* knockout (KO) in neuronal precursor cells impaired their maturation (53). Taken together, the evidence suggests that presence of FNDC5 is essential for the differentiation, proliferation, and maturation of neuronal cells.

*Fndc5* gene expression in neurons is under the regulation of PGC1α. Up-regulating *Ppargc1a* gene expression by adding forskolin (10 mM) into primary cortical neurons was accompanied by a significant elevation of *Fndc5* gene expression, whereas down-regulating *Ppargc1a* gene expression by nifedipine (5 mM) administration decreased *Fndc5* expression levels (52). Furthermore, forced *Ppargc1a* expression in primary cortical neurons led to a fourfold increase in *Fndc5* mRNA levels. Conversely, blocking of *Ppargc1a* gene expression with lentiviral-mediated shRNA KO decreased *Fndc5* mRNA expression by more than 40% (52). Additionally, the expression of *Bdnf* in brain is partly regulated by FNDC5. Wrann et al. (52) found that overexpression of *Fndc5* markedly increased *Bdnf* mRNA levels by fourfold, whereas *Fndc5* KO *via* lentiviral delivery of shRNA significantly reduced *Bdnf* expression in primary cortical neurons. Primary cortical neuronal cells cultivated with BDNF remarkably deceased *Fndc5* mRNA levels in a dose-repose pattern (52). More importantly, peripherally forced expression of *Fndc5* in the liver led to increased circulating irisin and up-regulated hippocampal expression of *Bdnf* (52). To determine the role of peripheral FNDC5/irisin in the brain, Lourenco et al. injected intravenously AdFNDC5 in mice to increase FNDC5/irisin overexpression (13). Intravenous administration of AdFNDC5 led to increment of both plasma and hippocampal FNDC5/irisin expression, and restored novel object recognition (NOR) memory defects in Alzheimer’s disease (AD) mice (13). These data indicate the capacity of circulating FNDC5/irisin entering into the brain to elevate FNDC5/irisin expression, and providing protection against memory impairment.

However, one concern about the beneficial effects of irisin on brain health is that it remains unclear how circulating irisin may pass through the blood-brain barrier (BBB). The BBB prevents the transport of big molecules from circulation to CSF, with only some small proteins capable of entering the central nervous system by passive diffusion (54). It is assumed that if peripheral irisin has effects on brain function, it needs a selective BBB transporter and a receptor on brain cells. It is known that irisin is only one of the exercise-stimulated proteins capable of promoting brain functions (9). Recently, a substitutive mechanism connecting exercise and improvement of learning and memory in rodents has been proposed (55). Lactate levels were elevated during exercise, which subsequently cross BBB and increase *Bdnf* expression through monocarboxylate transporter (MCT) 2 and upon the activation of Sirtuin 1 (SIRT1)/PGC1α/FNDC5/BDNF signalings (55).

### 3.2 Irisin and neurodegenerative diseases

To date, a vast body of literatures have demonstrated that irisin generates beneficial effects on neurodegenerative diseases. Lourenco et al. (13) investigated the capable of FNDC5/irisin alleviating synapse failure and memory impairment in AD. FNDC5/irisin levels were decreased in AD hippocampi and CSF, whereas there had no significant changes in plasma irisin. They knocked down *Fndc5 via* injecting brain with lentiviruses harboring two types of shRNA constructs. This caused impairment of hippocampal long-term potentiation (LTP) and memory during NOR task. Conversely, elevating *Fndc5* expression in hippocampus restored synaptic plasticity and memory in AD mice (13). Similar to the work of Wrann and colleagues, peripherally up-regulating FNDC5/irisin restored memory deficit, whereas blocking FNDC5/irisin expression in peripheral and brain inhibited the protective effects of exercise on synaptic plasticity and memory in AD murine models (13). Those data further supported the beneficial effects of peripheral FNDC5/irisin in improving brain function. Moreover, this link between irisin and its positive effects on brain function provides therapeutic implications of this protein and exercise in alleviating cognitive impairment in neurodegenerative diseases.

Wang et al. (56) cultivated astrocyte-conditioned medium with r-irisin for twelve hours, finding that r-irisin treatment protected the neurons from the toxicity of Amyloid-β peptides (Aβ). Moreover, irisin decreased the production of interleukin (IL)-6 and IL-1β from astrocyte culture and reduced the expression of cyclooxygenase 2 (*Cox2*) and phosphorylated protein kinase B (AKT) (56). Additionally, irisin attenuated nuclear factor kappa B (NF-κB) expression in astrocytes subjected to Aβ exposure by inhibiting phosphorylation and degradation of IκBα (56). A recent *in vitro* study investigated the potential role of FNDC5/irisin in the process of Aβ formation (57). Amyloid precursor protein (APP)-FNDC5 communication that occurs *via* connecting FNDC5 with a specific region between β- and α- secretase cleavage locations of APP decreased Aβ generation, resulting in a reduced production of Aβ_40_ and Aβ_42_ peptides in the cultured system (57). Other studies demonstrated that irisin were related to episodic memory and global cognition in individuals with high risk of dementia (58), and affected cognitive degradation in obese subjects at risk of AD (59).

Irisin benefits brain health partly due to its ability of inhibiting the inflammatory pathway. Both irisin and its precursor protein FNDC5 expression were significantly down-regulated whereas oxidative stress and reactive oxygen species (ROS)-NLR family pyrin domain containing 3 (NLRP3) inflammasome signaling were up-regulated in PC12 neuronal cells stimulated with oxygen-glucose deprivation (OGD) (60). The r-irisin treatment reversed OGD-induced oxidative stress and inflammation, but the irisin-related beneficial effects were inhibited by overexpression *Nlrp3*, suggesting that irisin alleviated OGD-caused neuronal impairment partly through blocking ROS-NLRP3 signalings (60).

## 4 Irisin-heart communication

### 4.1 Irisin in myocardial infarction

Myocardial infarction (MI) occurs when blood supply reduces or ceases to coronary artery of heart, resulting in injuries of heart muscle (61, 62). In US, approximately 550,000 adults experience first heart attack and 200,000 suffer recurrence of MI each year (63); it become a major source of the burden of diseases worldwide. The expressions of *Fndc5* mRNA and irisin protein have been detected in cardiomyocytes (35, 64), and irisin treatment increased cardiac regeneration and neovascularization (65) and enhanced diastolic volume, heart rate, and cardiac output (66). Current evidence suggests that irisin production is linked with the occurrence of MI and likely to become a potentially therapeutic target for MI. Cardiac ischemia frequently results in insufficient energy production, and lower irisin levels might benefit the maintenance of energy homeostasis by the suppression of browning white adipose tissue. In accordance with this notion, irisin production were found gradually reducing within 24 hours in a rat model of MI (67), and decreased expression of skeletal muscle *Fndc5* and reduced serum irisin levels were detected in rats with MI or cardiac ischemia-related heart failure (68–71).

Current evidence supports irisin as a therapeutic target for MI. Ischemia and reperfusion (I/R) and hypoxia-reoxygenation (HR) are usually applied as common models of MI *in vivo* and *in vitro* studies. Wang et al. (72) performed experiments with a rat model of heart I/R to determine whether systemic administration of irisin could protect against I/R-induced tissue injury. The treatment of r-irisin protected rat heart from I/R-induced injury in a dose-dependent manner. They further showed that r-irisin treatment reduced apoptosis of cardiomyocytes and oxidative stress by increasing superoxide dismutase (SOD) activity and restoring the mitochondria localization of SOD2 in cardiomyocytes (72). Liao and colleagues (73) demonstrated that, in a mice MI model, two-week r-irisin administration increased cardiac function, alleviated ventricular dilation, and decreased infarct size and fibrosis at four weeks after MI. The therapeutic effects are associated with the pro-angiogenic function and suppression of cardiomyocyte apoptosis *via* triggering extracellular signal-regulated kinase (ERK) pathways (73). Zhao et al. (74) reported a protective effect of irisin on H9C2 cells exposed to hypoxia/reoxygenation lesions partially *via* regulating histone deacetylase 4 (HDAC4). The dynamin-like GTPase optic atrophy1 (Opa1) overexpression could protect myocardial cells against hypoxia-linked impairment and increased cell survival through triggering autophagy. Irisin treatment stimulated Opa1-related autophagy, and protected cardiomyocytes from further damage following MI (75). Additionally, as a treatment strategy, r-irisin administration reduced myocardial infarct size, attenuated cell apoptosis, and preserved mitochondria function, as well as promoting SOD1 and p38 phosphorylation in an *in vitro* model of myocardial I/R injury (76).

### 4.2 Irisin in cardiomyocyte hypertrophy

Cardiomyocyte hypertrophy is a common outcome of long-term pressure/volume overload in a variety of cardiovascular diseases (77). Pathological situations, *e.g.* hypertension and heart attack, continuedly initiate cardiomyocyte hypertrophy and ultimately cause heart failure, meanwhile myocardial cell necrosis, fibrosis, fetal gene activation, disturbance of mitochondria function, and structurally sarcomeric alterations may occur (78). Among the signaling mechanisms that induce these responses, reprogramming of systemic and heart metabolism precede the progress of cardiomyocyte hypertrophy. Given the important role of irisin in regulation of metabolism, it is not surprised when several studies found the associations of irisin expression and the occurrence of cardiac hypertrophy (69, 71, 79). The changes in irisin may imply a damaged metabolic adaptation during the hypertrophic process and are associated with cardiomyocyte hypertrophy-induced cardiomyocyte death.

Both *in vivo* and *ex vivo* evidence indicates that FNDC5/irisin is involved in the overload/obesity-induced cardiomyocyte hypertrophy by affecting autophagy, inflammation, fibrosis, and oxidative stress (71, 79, 80). Several signaling pathways are suggested to involve the irisin-associated improvement of cardiomyocyte hypertrophy. Irisin prevents overloading-caused cardiac hypertrophy mainly *via* triggering defensive autophagy by stimulating Adenosine Monophosphate Activated Protein Kinase (AMPK)-Unc-51 like autophagy activating kinase 1 (ULK1) pathway (71). Moreover, irisin administration could improve overloading-caused cardiac dysfunction and myocardial hypertrophy *via* suppressing oxidative stress through downregulating AKT signaling activation (81). Yu and colleagues reported that cardiac and circulatory expressions of irisin were increased in cardiac hypertrophy mice, and the treatment of irisin is able to inhibit pathological cardiac hypertrophy and fibrosis through AMPK-mammalian target of rapamycin (mTOR) signaling pathways (79). In addition, Zhao et al. suggested that cardiomyocytes secreted irisin in an ADAM family dependent manner, and irisin administration improved overloading-caused cardiac hypertrophy and fibrosis and enhanced cardiac function by activating AMPK-mTOR signaling pathways (82).

## 5 Irisin-liver communication

### 5.1 Irisin in glucose and cholesterol metabolism

Liver is one target organ of the physiological action of irisin. Liver plays an important role in regulating gluconeogenesis, lip metabolism, inflammation, oxidation, *etc.* which are associated with hepatic diseases, including hepatitis, cirrhosis, non-alcoholic fatty liver disease (NAFLD), steatohepatitis (NASH), and liver cancer.

Liver regulates blood glucose concentrations by controlling glucose production, uptake, storage, and release *via* glycogen synthesis and degradation (83), and it is one major organ involving insulin resistance, a hallmark of metabolic syndrome and many of those patients eventually result in the onset of type 2 diabetes mellitus (T2DM) (84). In cultured hepatocytes with insulin resistance, r-irisin treatment (20nM) decreases gluconeogenesis *via* decreasing phosphoenolpyruvate carboxykinase (PEPCK) and glucose-6-phosphatase (G6Pase) expression through the phosphatidylinositol 3-kinase (PI3K)/AKT/Forkhead box protein O1 (FOXO1) signalings, and increases glycogenesis through PI3K/AKT/glycogen synthase kinase 3 (GSK3)/glycogen synthase (GS) signaling cascades (12, 85). Additionally, in diabetic obese C57BL/6 mice, the injection of r-irisin (0.5 µg/g body weight) caused a reduction in *Pepck* and *G6pase* expressions in the liver by stimulating the AMPK signaling pathway, and blocking of AMPK signaling decreased the influence of irisin on hepatocytic expressions of *Pepck* and *G6pase* (86).

Moreover, irisin is involved in hepatic cholesterol synthesis. Subcutaneous injection of r-irisin (12 nmol/d·kg body weight) induced a reduction in hepatic cholesterol in obese mice, and, in primary hepatocytes from lean and obese mice, r-irisin treatment remarkably reduced cholesterol concentrations by the inhibition of sterol regulatory element-binding transcription factor 2 (SREBP2) (87). The r-irisin administration prevented the palmitic acid (PA)-caused lipid deposition and lipogenic factors, acetyl CoA carboxylase (ACC) and fatty acid synthase (FAS), *via* inhibiting protein arginine methyltransferase-3 (PRMT3) in hepatocytes (37). Mo et al. has confirmed FNDC5/irisin to be a direct transcriptional target of constitutive androstane receptor (CAR), and the activation of CAR upregulates *Fndc5* expression in the liver (88).

### 5.2 Irisin and inflammation

Irisin can act as anti-inflammatory factor in the adipose tissue. Irisin alleviates adipose tissue inflammation partly *via* inhibiting macrophage polarization (89, 90). More interestingly, recent studies have proposed an anti-inflammatory action of irisin against SARS-CoV-2 infection. RNAseq-based data have revealed that irisin affects multiple genes related to the SARS-CoV-2 infection in human subcutaneous adipocytes (91), suggesting that the potential beneficial effects of irisin on COVID-19 outcomes are beyond its anti-inflammatory action on macrophages. In this regard, irisin inhibits SARS-CoV-2 entry points and the spike glycoprotein subunit S1-induced inflammatory cell death in human visceral adipocytes (92). Irisin also inhibits oxidative stress through reducing inflammatory biomarkers, including NF−κB, COX2, TNF and IL−6, and downregulating hepatocytic ROS generation. The effects are regulated *via* downregulating PRMT3 (37). Exogenous r-irisin treatment (250 μg/kg) promoted hepatic functions, decreased hepatocytic necrosis and apoptosis, and alleviated inflammation after liver I/R (93). Additionally, r-irisin treatment increased mitochondria function related *Ppargc1a* and *Tfam* expressions, and reduced oxidative stress *via* elevating UCP2 levels in liver I/R (93). Irisin ignited autophagy and promoted mitochondrial action by up-regulating telomerase function in aged liver cells, and then decreased the inflammatory responses, oxidative stress, apoptosis, and hepatic impairment in an aged murine liver I/R model (94). The effects of irisin on the increased telomerase activity is regulated by the inhibition of the phosphorylation of c-Jun N-terminal kinases (JNKs) during hepatic I/R (94).

### 5.3 Irisin and hepatocellular carcinoma

Additionally, irisin is also involved in the pathological process of human hepatocellular carcinoma (HCC). The levels of *Fndc5* mRNA in liver were upregulated in HCC patients compared to that in donors, and positively associated with the transcription factor sterol regulatory element-binding factor-1 (*SREBF1*), stearoyl-CoA desaturase (SCD1), *TNFα*, and *Il6* mRNA expressions in HCC patients (95). Another study observed a reduction of *Fndc5* expression in HCC tissues, and lower circulating irisin levels were related to high comprehensive complication index (CCI) marks after hepatectomy (96). Similarly, Shi and colleagues reported that irisin expression significantly increased in cancerous livers compared with those in the controls. And it upregulated cell proliferation, invasion, and migration partly *via* activating PI3K/AKT signalings, and decreased the cytotoxicity of doxorubicin in HepG2 cells (97).

## 6 Irisin-bone communication

The mechanical coupling of skeletal muscle and bone is obvious. Bone continually adjusts its mass and architecture to the changes of mechanical strain, and muscle contractions are essential for applying load to the bone. This mechanical perspective implies that muscle function declines resulting in reduced loading on bone and causing a reduced bone mass subsequently. Moreover, the two tissues also communicate in an endocrine manner in which muscle secretes myokines during exercise and regulates the physiological functions and pathological processes of bone (8, 15, 98). The discovery of irisin receptor–αV/β5 in bone cells provides further molecular evidence for the muscle-bone interaction through irisin signalings in a variety of physiological and pathological conditions (14).

### 6.1 Irisin and bone in animals

#### 6.1.1 Irisin and bone mass

Some studies have reported more interesting findings about injection of r-irisin enhancing bone mass and microstructure in mice. Colaianni et al. (99) measured the tibia bone of mice received four-time injections of 100 µg/kg r-irisin per week. They detected a significant increment of cortical bone mineral density (BMD) and strength. Applying X-ray imaging, r-irisin treatment elevated femoral and tibia bone density and bone circumference. Mice received r-irisin intervention showed elevated polar moment of inertia and bending strength. Moreover, the cortical bone expressed an elevated mRNA levels for osteopontin (*Osn*) (involved in bone formation) and a decreased sclerostin (SOST) (99). In another study, Levene et al. reported the administration of r-irisin increased BMD and restored impaired microstructure in ovariectomized rats *via* relieving inflammatory cytokines (TNF-α, IL-6, *etc.*) and up-regulating bone formation marker genes [such as Runt-related transcription factor 2 (RUNX2), B-cell lymphoma 2 (BCL2) and Nuclear factor erythroid 2-related factor 2 (NRF2)] (100). It is indicated that the positive effects of r-irisin on bone density and microstructure partly *via* enhancing osteogenic gene expression.

#### 6.1.2 Irisin and FNDC5-KO models

*Fndc5* KO is an important approach to the investigation of the effects of irisin on bone remodeling. Luo et al. (101) generated a global *Fndc5* KO murine model. They found that the lacking of irisin reduced bone mass and bone strength, and increased osteoclast numbers and RANKL cell surface expression in mice. The levels of IL-6 and tumor necrosis factor-α (TNF-α) were also enhanced in irisin-deficient mice (101). Taken together, it is suggested that irisin seem to involve in the mediation of bone homeostasis. In another study, Zhu and colleagues generated FNDC5/irisin specific ablation mice in osteoblastic lineage (Osx-Cre:FNDC5/irisin KO mice). It led to decreased *Fndc5* mRNA and irisin protein expressions in bone, reduced bone mass, and retarded bone formation and mineralization (102). These *Fndc5* KO mice also exhibited lower cortical BMD and trabecular bone volume in contrast to controls. However, the cortical bone surface/volume ratio accretion indicated thinner cortical bones (102). *Fndc5* KO mice exhibited decreased osteoblastic genes [*Runx2*, bone sialoprotein (*Bsp*), osterix (*Osx*), and alkaline phosphatase (*Alp*)] and enhanced osteoclastic genes [tartrate-resistant acid phosphatase (*Trap*), *Mmp9*, and *cathepsin K*]. Additionally, exercise did not benefit *Fndc5* KO mice by increasing bone strength and body weight loss, but treatment of r-irisin restored the exercise-related beneficial effects (102). However, Kim et al. reported that the *Fndc5* KO mice displayed elevated femoral trabecular BMD and better micro-structure (14). More interesting, *Fndc5* KO mice protected against ovariectomy exhibiting maintenance of bone mass and changed receptor activator of nuclear factor kappa-B ligand (RANKL) expression. In this study, the authors firstly demonstrated that irisin acted *via* the αV/β5 integrin receptor in osteocytes (14). Finally, they assumed that the action of irisin might like that of Parathyroid Hormone (PTH), acting to initiate both resorption and formation upon the pattern of r-irisin administration.

#### 6.1.3 Irisin and unloading models

Hindlimb unloaded animals were frequently used as a paradigm to investigate the microgravity impact on bone intravitally. The hindlimb-suspended mice showed accelerated femur bone loss, and decreased mineral deposition rates of trabecular and cortical bone (103). *Fndc5* together with the osteogenic marker mRNA levels were decreased. The activity of ALP and mRNA expressions of *Alp*, *Colia1* and *Fndc5* in primary osteoblasts were decreased under the condition of microgravity. The r-irisin administration (1 nM) for primary osteoblasts in the condition of simulated microgravity up-regulated gene expression of osteogenic markers, ALP activity, and mineralization (103). In a study by Kawao et al., hindlimb unloading decreased muscle cross-section area around tibia, lower leg muscle weights, and tibial trabecular bone density in mice (104). Hindlimb unloading also decreased *Fndc5* mRNA expression in the soleus muscle of mice, and it was positively related to trabecular BMD in the tibia and negatively correlated with *Rankl* mRNA levels in the tibia of mice (104). On the contrary, mechanical loading elevated *Fndc5* mRNA levels, which could be inhibited by blocking bone morphogenetic protein (BMP) and PI3K signalings. It is suggested that irisin may involve in the mechanical unloading-caused muscle loss and bone mass reduction, and BMP and PI3K pathways probably involve in those pathophysiological conditions. Metzger et al. reported that weekly three times of r-irisin treatment (18 ng/mL) increased bone homeostasis and bone formation rate in hindlimb unloaded rats, and decreased osteoclast differentiation and production of TNF-α, IL-17, RANKL and SOST in the suspended hindlimb (105). These data suggested that r-irisin treatment protect unloaded rats from disuse-induced bone loss through reducing pro-inflammatory cytokines and bone turnover.

#### 6.1.4 Irisin and other conditions

Irisin can also inhibit bone cell apoptosis and then improves ovariectomy-induced bone loss. Xu and colleagues showed that r-irisin administration significantly enhanced trabecular thickness, number, and BMD in estrogen-deficient rats (106). The expressions of *Runx2*, osteocalcin (OC), *Bcl2* and *Nrf2* were higher whereas those of caspase 3 and *Nlrp3* were lower in the r-irisin intervention group. Irisin increases Nrf2, decreases NLRP3 inflammasome, and reduces inflammatory cytokines, thus mitigating osteoblast apoptosis in estrogen-deficient rats (106).

Inflammatory bowel disease (IBD) is a group of inflammatory conditions of the colon and small intestine that not only leads to gastrointestinal dysfunction but inflammation-related bone loss (107). Twice weekly treatment of r-irisin (18 ng/mL) increase gut and bone properties through alleviating inflammation and reconstituting structure. In bone, IBD increased osteoclast surface and decreased bone formation, and r-irisin treatment reversed this phenotype (107). Furthermore, r-irisin treatment also decreased the levels of TNF-α and RANKL in osteocytes in bone (107). Sprague–Dawley rats with severe IBD received twice weekly r-irisin treatment for 3 weeks showing increased osteogenic rate, reduced osteoclastic resorption, and down-regulated pro-inflammatory cytokines (108).

### 6.2 Irisin and bone in humans

Circulating irisin concentrations were positively associated with Oc and cyclophosphamide (CTX), and negatively related to Dickkopf-related protein 1 (DKK-1) circulating concentrations (109). Circulating irisin levels were inversely associated with age, PTH, and creatinine levels (110). The evidence implies that irisin might act as a predictor for bone statues and plays a determinant role in bone metabolism. Irisin is correlated to low bone mass in postmenopausal women. Circulating irisin concentrations were markedly decreased in osteoporotic postmenopausal females (111). Serum irisin concentrations were decreased whereas c-reactive protein levels were increased in osteoporotic women, indicating that irisin may act as a modulator in the pathogenesis of osteoporosis. Colaianni et al. reported that circulating irisin concentrations were lower in osteopenic/osteoporotic patients in contrast to healthy individuals, and positively associated with femoral and vertebral BMD in patients with total hip or knee replacement (112).

Zhang et al. reported that older Chinese men with low bone mass had decreased circulating irisin concentrations compared with age-matched controls, and circulating irisin concentrations were an independent predictor for BMD (113). Yan et al. demonstrated that, as an independent factor of bone density, irisin account for approximately 17.8% and 22.5% of femoral neck and lumbar spine BMD, respectively (114). In multiple linear regression, Anastasilakis et al. found that the risk of osteoporotic fracture and serum PTH levels were inversely correlated with irisin concentrations (110). Irisin was positively correlated with *FNDC5* gene expression in muscle biopsies as well as *Oc* mRNA in bone biopsies, and FNDC5 positive fibers were closely linked to BMD of total femur and femoral neck, respectively (112). Furthermore, irisin directly affected the senescence marker p21, which was highly expressed in the bone biopsies of osteoporotic patients, by down-regulating the *p21* mRNA expression in osteoblasts (112). Those data suggest that irisin is an independent factor for bone density, bone biomarkers, and the occurrence of fracture.

## 7 Signaling pathways for irisin-exercise-organ communications

### 7.1 PGC1α–irisin–BDNF pathway in exercise and brain function

Irisin is involved in the neuroprotective effects of exercise against ischemic attack. Li and colleagues (115) reported that circulating irisin concentrations and muscular FNDC5 protein levels reduced after ischemic attack, and its concentrations were negatively correlated to cerebral infarction size and the neurological function deficient scores. Interestingly, irisin levels in skeletal muscle increased after physical exercise, and 2-week exercise training significantly reduced brain infarction volume (115). This beneficial impact of exercise was weakened by irisin-blocking agents. Similarly, exercise also decreased *TNFα* and *Il6* expressions in the peripheral infarct area of cerebral tissues in the middle cerebral artery occlusion mice, which was inhibited by administration of irisin antibody (115). Injecting 6-hydroxydopamine into the medial forebrain bundle (MFB) in rats induced impairments in behavioral and a remarked decrease in the expressions of *Ppargc1a*, *Fndc5*, and *Bdnf* mRNA in both the striatum and the hippocampus compared with the sedentary controlled rats, whereas those negative changes were reversed by 16-week exercise intervention (116). It is indicated that physical exercise can alleviate neurodegeneration and learning deficiency by promoting the cerebral expression of certain effective and relevant factors.

Increasing evidence from *in vivo* and *ex vivo* experimental animal models has suggested that the favorable impact of exercise on memory and neural plasticity is mediated by irisin. A daily swimming programme (1h per day, 5days per week for 5weeks) protected mice from amyloid-β oligomers (AβOs)-induced memory impairment and decrease in the expressions of *Fndc5* mRNA and irisin protein in mouse hippocampus (13). However, exercise-induced improvement of LTP was inhibited by intracerebroventricular injection of a lentiviral vector harboring *Fndc5* shRNA (13). More intriguingly, intraperitoneal injection of FNDC5-inhibiting antibody blocked the neuroprotective effects of exercise against the synaptic plasticity and memory damage induced by AβOs. Similarly, exercise protected against the reduction of hippocampal *Fndc5* expression induced by AβOs, but the positive effects were blocked by peripheral administration of anti-FNDC5 (13). Additionally, elevating serum irisin levels *via* overexpression of irisin in liver *via* adenoviral vectors led to increment in cerebral expression of irisin sufficient to protect against cognitive impairment and neural pathology in AD mice (117). Those data suggest that circulating irisin could cross the BBB and modulate the neural protective effects of exercise on neural plasticity and memory in AD.

**Figure 2:**
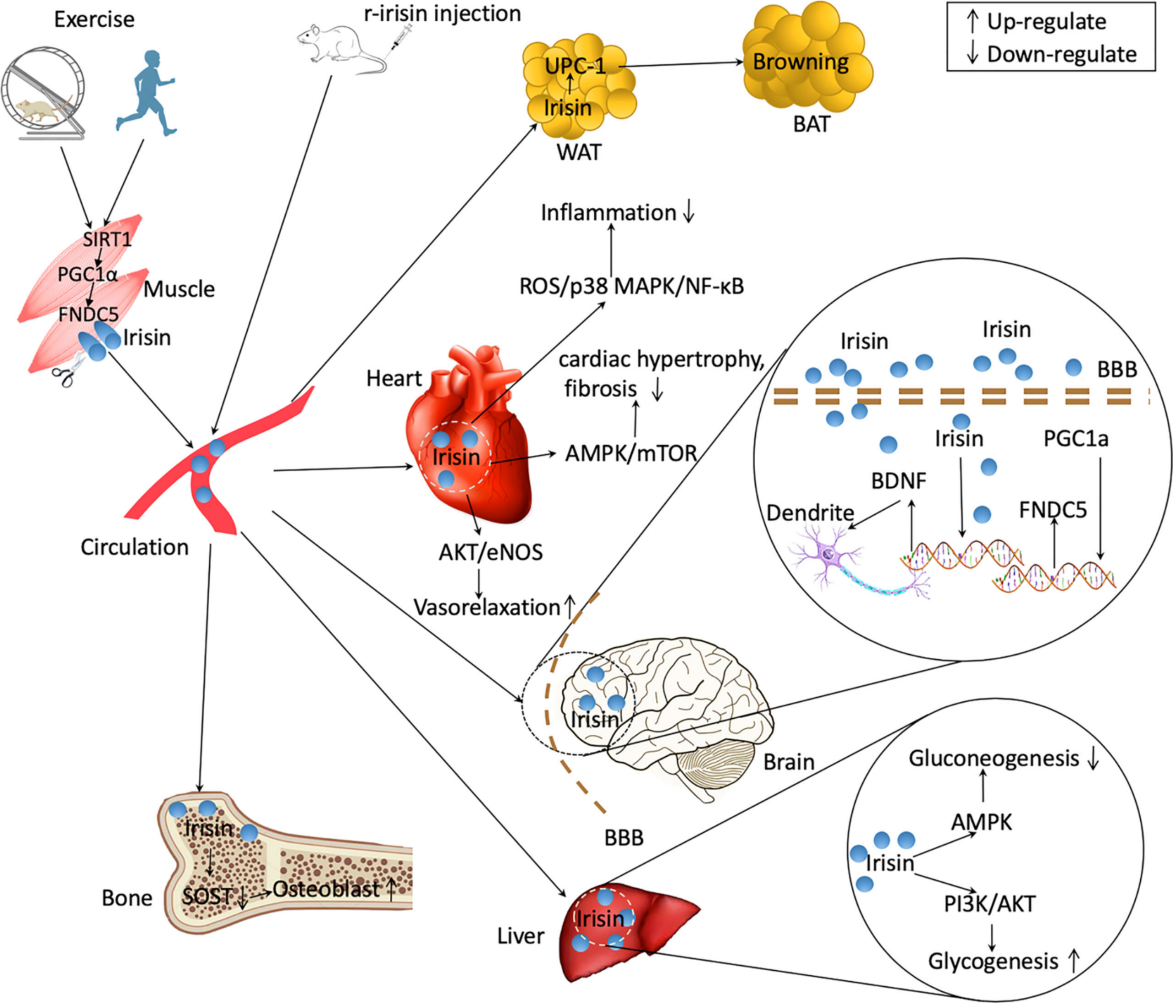
Irisin regulating the communications between organs. Exercise upregulates the expression of peroxisome proliferator-activated receptor γ coactivator 1α (PGC1α) in skeletal muscle in mice and humans by upregulating Sirtuin 1 (SIRT1). *Ppargc1a* expression in muscle induces an increased expression of fibronectin type III domain-containing protein 5 (FNDC5), which is released as irisin into the circulation after cleavage. Some organs such as brain and heart also produce irisin in response to exercise. Peripherally treated recombinant irisin (r-irisin) can enter multiple organs and exert physiological effects. Irisin can lead to browning of white adipose tissue (WAT) increasing energy expenditure. In brain, irisin upregulates brain-derived neurotrophic factor (BDNF) expression and increase the plasticity of dendrite. Irisin promotes osteoblast differentiation by decreasing sclerostin (SOST) signalings. In liver, irisin decreases gluconeogenesis through adenosine monophosphate activated protein kinase (AMPK) signaling and increases glycogenesis *via* phosphatidylinositol 3-kinases (PI3K)/protein kinase B (AKT) pathways. Irisin also decreases inflammation, mitigates cardiac hypotrophy and fibrosis, and increase vasorelaxation. BAT, brown adipose tissues. BBB, brain-blood barrier. ROS, reactive oxygen species. NF-κB, nuclear factor kappa B. eNOS, endothelial NO synthase.

Exercise-related improvement of brain function is linked to the irisin-induced *Bdnf* expression. Choi et al. demonstrated that the exercise-stimulated neurogenesis in adult hippocampus was correlated with the increment of cerebral *Fndc5* and *Bdnf* expressions, subsequently promoting cognitive function in AD mice (118). As one of target genes of *Ppargc1a*, the expression of *Fndc5* is regulated by *Ppargc1a*. Wrann et al. (52) reported that exercise induced increased *Ppargc1a* expression leading to increment in hippocampal *Fndc5* gene expression in mice. Subsequently, the changes in *Fndc5* gene expression affect *Bdnf* expression; overexpressing *Fndc5* in primary cortical neurons stimulated increment of BDNF levels, whereas KO of *Fndc5 via* siRNA led to decrease in *Bdnf* expression. Moreover, adenoviral delivery of FNDC5 to liver led to elevation of circulating irisin and increased hippocampal *Bdnf* gene (52). Those data suggest the existence of muscle–brain crosstalk during exercise, which is regulated partly through the activation of PGC1α/FNDC5/BDNF signaling pathway (**Figure 2**).

### 7.2 Irisin-p38/MAPK-GLUT4 axis in exercise and insulin resistance

As an energy metabolism modulator, irisin is engaged in the impact of exercise on insulin action and glucose metabolism. Six-week treadmill training significantly increased serum concentrations of irisin and decreased serum insulin and glucose levels in mice (119). Serum irisin concentrations were negatively correlated to homeostatic model assessment of insulin resistance (HOMA-IR) (119). Korkmaz et al. (120) determined the relationship between irisin and glucose metabolism in exercised overweight/obese subjects (n = 144) with impairment of glucose regulation. Compared to the sedentary group, a program of aerobic type Nordic walking showed elevated plasma irisin concentrations. Training-related changes in muscular *Fndc5* gene were negatively associated with the change in HB-A1c-IR, and circulating irisin levels were positively correlated with insulin concentrations following a 2-hour oral glucose tolerance test (120).

More interestingly, secretion of irisin during exercise might be up-regulated by cold exposure to increase brown fat thermogenesis. Ulupinar et al. (121) showed that aerobic exercise in a cold environment induced greater irisin secretion. Lee et al. (122) reported that cold exposure significantly elevated circulating irisin levels and irisin increment was proportional to shivering magnitude, similar to exercise-induced secretion. The authors pointed out that exercise-stimulated irisin secretion might represent a thermogenic, cold-activated endocrine axis, serving to augment brown fat thermogenesis (122). Those findings suggested that the combination of cold exposure with exercise increased irisin secretion to promote energy expenditure.

The ability of exercise increasing glucose uptake in skeletal muscles is regulated by increasing expression of glucose transporter 4 (GLUT4) (123). In a programme of 45-minute treadmill running (30 m/min, 15% grade), the levels of *Glut4* gene expressed on the membrane of skeletal muscle cells in rats increased by 2.5 times (124). GLUT4 is one of the most essential glucose transporters, and its action is closely correlated to insulin action (125). Exercise increases the content and trafficking of GLUT4 in muscle cells in part through up-regulating irisin production. Eight-week interventions of high-intensity interval training (HIIT) or moderate-intensity continuous training (MICT) remarkably increased the expression of *Fndc5* gene in the soleus muscle of rats (126). In the meantime, the *Glut4* gene expression in soleus muscle were also remarkably increased, and MICT rats exhibited greater effects than those of HIIT rats (126). The capacity of irisin up-regulating *Glut4* expression in muscle is assumed partly *via* activating p38/mitogen-activated protein kinase (MAPK) signalings. Exercise significantly up-regulated the expressions of *Ppargc1a* mRNA and *Fndc5* mRNA in murine skeletal muscle (46, 127), and simultaneously *p38* and *Mapk* mRNA levels were also markedly elevated (128). Irisin can promote the translocation of GLUT4 to the muscle cell membrane by phosphorylation of p38/MAPK, and subsequently increase up-taking glucose *via* skeletal muscle (129). Taken together, it is suggested that exercise improves insulin resistance partly *via* activating irisin/p38/MAPK/GLUT4 signaling pathway (**Figure 2**).

### 7.3 FNDC5/Irisin-PINK1/Pakin-LC3II/I-P62 pathway in exercise and heart health

Exercise is a strong stimulator for the production of FNDC5/irisin in cardiac tissue (130–132), and the exercise-induced changes in irisin are associated with heart diseases (10, 133). Aydin et al. reported that irisin is highly presented in myocardium of both sedentary and exercise young rats, and it increased particularly in the connective tissues of heart after acute exercise (134). In accordance with this, irisin concentrations increased both in the supernatants from cardiac tissues of exercised rats and in circulation after exercise intervention (134). Serum irisin levels significantly increased in infarct rats received exercise training compared with those without exercise intervention (44.28 ± 2.20 vs. 30.70 ± 2.66 ng/mL) (135). Meanwhile, the levels of caspase-3 expression and the percentage of collagen deposition were increased in infarct rates whereas those negative outcomes were alleviated after exercise intervention. Furthermore, the changes in caspase-3 expression and collagen deposition were negatively correlated with serum concentrations of irisin (135). Twelve weeks of daily1-h treadmill training induced greater increase in serum irisin levels and murine cardiac function, including the ejection fraction (12%), fractional shortening (17%), cardiac output (29%), stroke volume (21%), end-diastole volume (34%), and end-systolic volume (58%) (136). Those data suggest that the exercise-stimulated irisin secretion helps to improve cardiac function and ameliorate the severity of heart diseases.

Exercises enhanced remarkably cardiac *Fndc5* expression, and meantime PTEN-induced kinase 1 (PINK1)/Parkin pathway and mitophagy activity were also up-regulated which led to increased antioxidant function and improved cardiac function (137). PINK1/Parkin signaling is a key regulatory pathway for mediating mitophagy action, and the up-regulation of PINK1/Parkin activates mitophagy activity *via* stimulating mitochondrial microtubule-associated protein light chain 3 (LC3II/I) and SQSTM1/p62 (P62) pathway (138). Consequently, increased mitophagy activity helps to alleviate MI *via* improving hypoxia, metabolic disturbance, and cardiac dysfunction (139). It is indicated that exercise decreased oxidative stress and promoted heart function in part through triggering FNDC5/Irisin-PINK1/Pakin-LC3II/I-P62 signalings (**Figure 2**).

### 7.4 Irisin-SOST pathway in exercise and bone

The efficacy of physical activity to increase bone health is due to the capacity of continuously generating mechanical loading upon bone tissues (140). Mechanical strain could be sensed by osteocytes and is transferred from physical stimuli into electronic signalings to regulate osteoblast and osteoclast activity. SOST, one master protein secreted by osteocytes, acts as an inhibitor of osteoblast differentiation *via* blocking Wnt/β-catenin signalings (141, 142). People who have more physical activities shows lower level of SOST, and regular exercise intervention induced a significant reduction of SOST level (143, 144). Circulating irisin and SOST are highly negatively correlated (145). Four-week treatment of r-irisin induced a significant increment in cortical BMD (7%), and the cortical bone contained higher level of *Osn* mRNA and lower level of *Sost* expression (99). In another study, Colaianni and colleagues (145) suspended the hindlimb of mice, which led to bone density reduction. Four-week unloading induced ~4% and ~40% reduction of cortical and trabecular BMD in femurs (145). Weekly administration of r-irisin (100µg/kg) protected against the cortical and trabecular bone loss *via* decreasing *Sost* expression and increasing osteoprotegerin expression (145) (**Figure 2**). However, Kim and colleagues (14) reported that r-irisin treatment up-regulated *Sost* expression both *in vivo* and *ex vivo* experiments. Given the fact that each dose (1 mg/kg per day) is nearly 10 times higher than the dose used by Colaianni and colleagues (100µg/kg per week), the amount of irisin and durations or frequencies of intervention may account for the controversies. Taken together, the current studies suggested the beneficial effects of exercise on bone metabolism partly *via* irisin/SOST pathway.

## 8 Perspectives

To date, compelling studies have suggested interesting roles for irisin, initially in muscle and adipose, and subsequently extending to bone, heart and brain. It is obvious that irisin lies on a center of the intercommunications between organs or tissues. As an “exercise hormone”, irisin promotes energy consumption by browning of white fat tissues during exercise. However, there remains some concerns needing to be addressed by future studies. Although αVβ5 integrin has been identified as the irisin receptor in osteocytes, adipocytes, and enterocytes (14, 146), it needs to be confirmed in other organs or tissues, such as brain, liver, heart, *etc.* Any progress in this field will improve our understanding of the signalings of irisin regulating the interactions between exercise and organs or tissues. However, the findings of some studies have been questioned for using questionable antibody-based assays; therefore, it will help improve the accuracy in determination of circulating irisin using the valid ones. Fortunately, *in vivo* or *ex vivo* exposure to irisin in cell culture or rodent models has provided a reliable approach to determine its physiological and pathological roles in health and diseases. The use of gain-of-function or loss-of-function models by injection of r-irisin or *Fndc5* deletion provides a powerful tool to address controversies and to determine the mechanism of irisin-induced beneficial effects.

## Author contributions

RZ reviewed the clinical evidence, performed the data synthesis, interpreted findings, wrote the manuscript, and led the work as a whole.

## Funding

This work was supported by Natural Science Foundation of Jiangsu Province (BK20201435). The funder has no role in writing manuscript and determining the submission of manuscript.

## Conflict of interest

The author declares that the research was conducted in the absence of any commercial or financial relationships that could be construed as a potential conflict of interest.

## Publisher’s note

All claims expressed in this article are solely those of the authors and do not necessarily represent those of their affiliated organizations, or those of the publisher, the editors and the reviewers. Any product that may be evaluated in this article, or claim that may be made by its manufacturer, is not guaranteed or endorsed by the publisher.
